# Remotely sensed environmental measurements detect decoupled processes driving population dynamics at contrasting scales

**DOI:** 10.1002/ece3.10358

**Published:** 2023-08-02

**Authors:** Avril M. Harder, Mekala Sundaram, Lana L. Narine, Janna R. Willoughby

**Affiliations:** ^1^ College of Forestry, Wildlife and Environment Auburn University Auburn Alabama USA; ^2^ Department of Integrative Biology Oklahoma State University Stillwater Oklahoma USA

**Keywords:** fitness, Landsat, monitoring, population dynamics

## Abstract

The increasing availability of satellite imagery has supported a rapid expansion in forward‐looking studies seeking to track and predict how climate change will influence wild population dynamics. However, these data can also be used in retrospect to provide additional context for historical data in the absence of contemporaneous environmental measurements. We used 167 Landsat‐5 Thematic Mapper (TM) images spanning 13 years to identify environmental drivers of fitness and population size in a well‐characterized population of banner‐tailed kangaroo rats (*Dipodomys spectabilis*) in the southwestern United States. We found evidence of two decoupled processes that may be driving population dynamics in opposing directions over distinct time frames. Specifically, increasing mean surface temperature corresponded to increased individual fitness, where fitness is defined as the number of offspring produced by a single individual. This result contrasts with our findings for population size, where increasing surface temperature led to decreased numbers of active mounds. These relationships between surface temperature and (i) individual fitness and (ii) population size would not have been identified in the absence of remotely sensed data, indicating that such information can be used to test existing hypotheses and generate new ecological predictions regarding fitness at multiple spatial scales and degrees of sampling effort. To our knowledge, this study is the first to directly link remotely sensed environmental data to individual fitness in a nearly exhaustively sampled population, opening a new avenue for incorporating remote sensing data into eco‐evolutionary studies.

## Graphical abstract

We leveraged publicly available remote sensing data to provide new context for detailed historic demographic data from a banner‐tailed kangaroo rat (*Dipodomys spectabilis*) population. Specifically, we were able to identify apparently decoupled effects of surface temperature on individual fitness and population size. To our knowledge, this study is the first to directly link remotely sensed environmental data to individual fitness measures, opening a new avenue for incorporating remote sensing data into eco‐evolutionary studies.

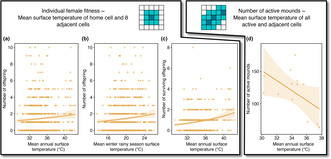

## 1 INTRODUCTION

Understanding the environmental drivers of population stability and fluctuations is critical for effective natural resource management. However, developing this understanding can require information about ecosystems and land cover at scales and sampling frequencies that are impractical to collect from field efforts alone. Beginning with the launch of the Landsat 1 satellite in July 1972, the National Aeronautics and Space Administration/U.S. Geological Survey Landsat Program has consistently provided medium spatial resolution satellite imagery of Earth's surface, with free and open access since 2008 (Wulder et al., 2022). Its data products have contributed to a rapid expansion of interdisciplinary research that relies on ecological knowledge and remote sensing data to describe a variety of patterns, including tracking loss of wetland habitat, detecting shifts in forest canopy composition, and monitoring shifts in phenological cycles (Vogelmann et al., 2016). Much of this work is forward‐looking, seeking to describe how natural systems evolve as climate change progresses and to construct relevant projections, but historical remote sensing data can also be used to add new dimensions to datasets lacking contemporaneous environmental measurements (e.g., Boult et al., 2018; Ndegwa Mundia & Murayama, 2009; Rossi & Leiner, 2022). Herein, we combine remote sensing and weather modeling data with previously collected demographic data to describe environmental factors influencing various components of population dynamics.

Our focal population of banner‐tailed kangaroo rats (*Dipodomys spectabilis*) has been the subject of myriad studies, including investigations of mate choice patterns, genetic adaptation to arid environments, philopatry and dispersal, and many other eco‐evolutionary dynamics (Busch et al., 2009; Jones et al., 1988; Marra et al., 2012; Waser & DeWoody, 2006). These studies were largely based on detailed demographic and genetic sampling, including precise home mound locations for nearly all individuals in the population and a nearly complete pedigree linking parents and offspring (Waser & Hadfield, 2011; Willoughby et al., 2019). Analysis of this pedigree has previously shown that genetic variables, including degree of individual inbreeding or relatedness between mates, explain a portion of individual fitness, but individual birth year (i.e., non‐genetic or environmental factors) accounted for a relatively larger proportion of variation in individual fitness (Willoughby et al., 2019).

To test which environmental characteristics contribute to these interannual differences in fitness, we used Landsat 5 Thematic Mapper (TM) images to obtain surface temperature data and three other descriptive indices via the Tasseled Cap Transformation (Tasseled Cap brightness, greenness, and wetness) (Kauth & Thomas, 1976). Tasseled Cap values can be used to describe variation in soil moisture content, ground cover type, and plant communities, with previous practical applications including assessing impacts of natural disasters, tracking shoreline changes, and charting the progress of desertification (Mostafiz & Chang, 2018; Shamsuzzoha & Ahamed, 2023; Zanchetta et al., 2016). We used these data alongside modeled precipitation and temperature data to summarize the environment of this population over 13 years. We analyzed these data in conjunction with demographic data at three different scales representing three distinct levels of field sampling effort—(i) individual microhabitat vs. individual fitness, (ii) population‐scale macrohabitat vs. population fitness, and (iii) population‐scale macrohabitat vs. population size—to test the suitability of remote sensing data for describing the effects that specific environmental variables can have on population dynamics at different scales. Because populations of banner‐tailed kangaroo rats have been the subjects of numerous ecological and evolutionary studies over several decades, we were able to compare the patterns observed in our results against inferences drawn from prior field‐based studies.

Previous studies of *D. spectabilis* and other heteromyid rodents have described positive relationships between the amount of habitat openness and survival or population size, perhaps because openness facilitates easier detection of or evasion maneuvers against predators or because higher quality food sources tend to grow in such habitats (Bowers et al., 1987; Germano et al., 2001; Waser & Ayers, 2003). We therefore expected to see a positive relationship between the Tasseled Cap brightness index and individual and population fitness, as brightness can indicate the ratio of open soil to plant cover (Crist & Cicone, 1984). We also expected to see a positive effect of precipitation and the Tasseled Cap wetness index—a measure sensitive to soil and vegetative moisture, but primarily characterizing soil moisture (Crist & Cicone, 1984)—on fitness, as increasing water availability may translate into increased food resources (Brown & Zeng, 1989; Munger et al., 1983). Subsequent increases in these resources may be captured by the Tasseled Cap greenness index, a measure shown to be correlated with leaf area index and vegetation biomass (Crist & Cicone, 1984). Specifically, we expected that higher greenness measures in the rainy seasons preceding breeding would lead to increased fitness, as previous studies have found lagged positive responses in rodent biomass or abundance to increased primary productivity (Ernest et al., 2000; Hernández et al., 2005; Previtali et al., 2009; Schooley et al., 2018). Finally, we anticipated that surface and air temperature measures would be negatively correlated with fitness, as increasing surface temperature corresponds to decreasing survival for *D. spectabilis* populations in the Chihuahuan Desert (Moses et al., 2012).

Although other studies have drawn important new ecological inferences by linking remotely sensed environmental measurements to approximations or correlates of fitness (where fitness is defined as the number of offspring produced by a single individual), such as apparent survival (Moses et al., 2012; Ward et al., 2018) or clutch size and fledging success (Regos et al., 2022; Riggio et al., 2023), ours is the first to use direct assessments of individual fitness as response variables. Specifically, the identification of parent–offspring pairs via genetic analysis allows for inclusion of adult individuals known to be alive but producing zero offspring within a year and for linking observations of specific individuals across years. Herein, we leverage this extensive demographic dataset to test our ecological predictions and, through these analyses, develop new ecological hypotheses regarding drivers of banner‐tailed kangaroo rat population dynamics. Overall, we demonstrate that, in the absence of locally collected environmental data, remote sensing data can be used to draw novel inferences and generate new questions regarding fitness and population dynamics at multiple spatial scales and degrees of sampling effort.

## 2 METHODS

### 2.1 Study system

**FIGURE 1:**
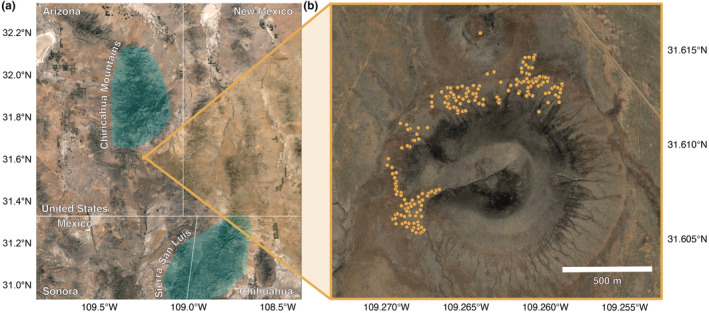
(a) Map of the area surrounding the study site, which is situated in Arizona near the New Mexico and Mexico borders. The site is located just southeast of the Chiricahua Mountains. (b) Map of the study site with all mounds included in this study marked with points. The mounds are located on primarily flat areas surrounding a cinder cone.

The study site is located in the Madrean Archipelago ecoregion, which comprises the Sky Islands—forested mountains interspersed among broad, flat desert scrub, and grasslands. The Chiricahua Mountains lie just to the north and west of the site, which is situated around a volcanic cinder cone surrounded by flatlands approximately 35 km southwest of Portal, AZ (31°36′27″ N, 109°15′48″ W) (Figure 1a). Annual precipitation patterns typically include a summer rainy season from July to August (which supplies 50% of total annual precipitation) and a second, less intense winter rainy season from December to March (Adams & Comrie, 1997). The study area is primarily desert grassland, with rare to occasional half‐shrubs and forbs present (Jones et al., 1988; Waser & Ayers, 2003).

Banner‐tailed kangaroo rats rely on these plant communities for food, caching seeds in large mounds (1–3 m in diameter) constructed for food storage, reproduction and protection from predators and harsh environmental conditions (Edelman, 2011; Kay & Whitford, 1978). Each mound is typically occupied and defended by a single individual, with the exception of females and their dependent offspring (Schroder, 1979). When the offspring are between 2 and 7 months old, they disperse from their natal mounds to nearby vacant mounds to establish individual territories (Jones, 1984; Waser et al., 2006). Exceptions to typical dispersal patterns may occur in years of high population densities, wherein individuals are more likely to remain in their natal mound to reproduce than to disperse to a new location (Jones et al., 1988; Waser & DeWoody, 2006). Mating typically occurs between December and March with females producing 1–2 litters of 1–3 offspring per year (Jones, 1984). Individuals typically live up to 4 years, often producing offspring during the first mating season of their lives.

### 2.2 Banner‐tailed kangaroo rat data collection

Our banner‐tailed kangaroo rat demographic data was collected from a population monitored by Waser et al. from 1990 through 2007 (Sanderlin et al., 2012; Skvarla et al., 2004; Waser & Jones, 1991). Twice annually, three traps were placed around each active mound on three consecutive nights, resulting in near‐exhaustive population sampling (98% median capture probability for adults; 93% for juveniles; Skvarla et al., 2004). Each captured individual was uniquely marked with ear tags and sex and mound‐specific capture location were recorded. It was also noted whether the individual was a juvenile (i.e., born in that year) or an adult. Ear tagging and subsequent recapture allowed individuals to be tracked across the landscape from year to year, and pinna biopsies were taken for genetic characterization. Biopsy samples were genotyped at nine polymorphic loci (Busch et al., 2009; Waser et al., 2006) and the resulting data were used alongside trapping records to construct a pedigree for the population (Waser & Hadfield, 2011; Willoughby et al., 2019). Briefly, Waser and Hadfield (2011) used MasterBayes to build the pedigree, with parental assignment probabilities influenced by trapping location and parent/offspring genotypes (see Willoughby et al., 2019 for details).

### 2.3 Environmental data curation and transformation

We downloaded all available Landsat 5 TM Collection 2 Level 2 images for our study site from 1989 to 2005. Our site was covered by both paths 34 and 35 in row 38 at 30‐m spatial resolution. All images were processed and analyzed in R v4.0.3 (R Core Team, 2020). Because of the small size of our site relative to the footprint of a Landsat 5 scene, each image was cropped to a 2100 m × 2750 m extent using the *raster* package prior to further processing (Hijmans, 2022). We manually reviewed the cropped natural color image for each scene to verify absence of clouds or any other source of error.

**FIGURE A1:**
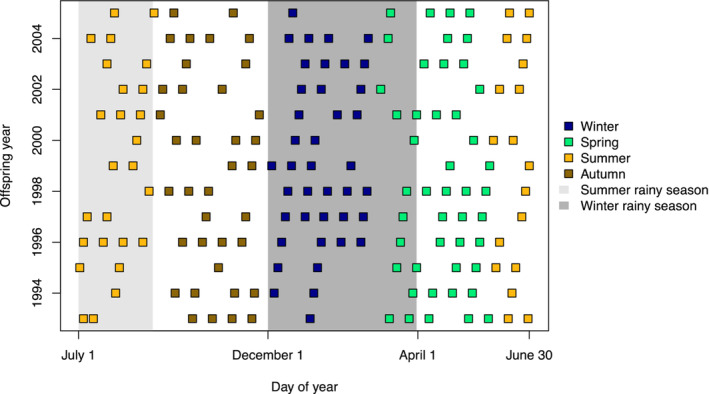
Temporal distribution of retained Landsat 5 scenes (*n* = 167) across rainy seasons and meteorological seasons used for equalizing means within each year. Note that the *y*‐axis indicates offspring year or the year in which a cohort of offspring was produced. Therefore, the environmental data used to predict the number of offspring produced in year *t* covers July 1 in year *t* − 1 through June 30 in year *t* (i.e., the first date included in this plot is July 1, 1992, and the last date included is June 30, 2005).

For each of the surface reflectance bands, we applied the multiplicative scale factor (0.0000275) and additive offset (−0.2) specified in the Landsat 4–7 Collection 2 Level 2 Science Product Guide (U.S. Geological Survey, 2021, pp. 4–7). We also converted the surface temperature band to Kelvin (and later to degrees Celsius) using a multiplicative scale factor of 0.00341802 and an additive offset of 149. Using the *spectralIndices* function in the *RStoolbox* package, we calculated three Tasseled Cap indices for all images: Tasseled Cap brightness, greenness, and wetness (Crist, 1985; Leutner et al., 2019). To check for biased values with respect to path number, we plotted the mean value of each index per scene (i.e., timepoint) over time. Across all years examined, values calculated from path 34 were consistently higher than temporally adjacent values calculated from path 35, leading us to rely exclusively on path 35 scenes for downstream analyses. We also limited the dataset to scenes collected from 1993 to 2005 due to limited observations available in 1989–1992, leaving 167 scenes (Figure A1; Table A1). All cell values across all years were *z*‐transformed within each Tasseled Cap index. After observing intra‐ and interannual patterns for these four variables, we calculated pairwise Pearson correlation coefficients using the *cor* function in R.

To link the remote sensing data to specific kangaroo rat mounds, we used GPS coordinates recorded for 188 mounds to assign them to corresponding cells in the raster. For 26 mounds, no GPS coordinates were available, but all mounds had been mapped during the original surveys using a custom coordinate system (units in meters) covering the study site (i.e., the position for each mound was recorded against a single reference point). Using the known coordinates for 188 mounds, we overlaid the meter‐based locations for all mounds onto the raster and manually assigned the mounds lacking GPS coordinates to cells in the raster. In total, we assigned 214 mounds to raster cells (Figure 1b).

We also obtained precipitation totals and minimum, mean, and maximum temperatures from the Parameter‐elevation Regressions on Independent Slopes Model (PRISM) at 4‐km resolution (PRISM Climate Group, Oregon State University, 2009). The PRISM model incorporates a digital elevation model and other spatial datasets to calculate gridded estimates of multiple climatic parameters, while accounting for the effects of terrain on precipitation (Daly et al., 1997, 2008). We used these estimated daily precipitation totals (mm) and minimum, mean, and maximum temperatures (°C) to calculate annual and seasonal means for the population‐level analysis, as a single value for each PRISM variable was available for the entire population (see below).

### 2.4 Data summarization and statistical analyses

#### 2.4.1 Individual fitness

**FIGURE 2:**
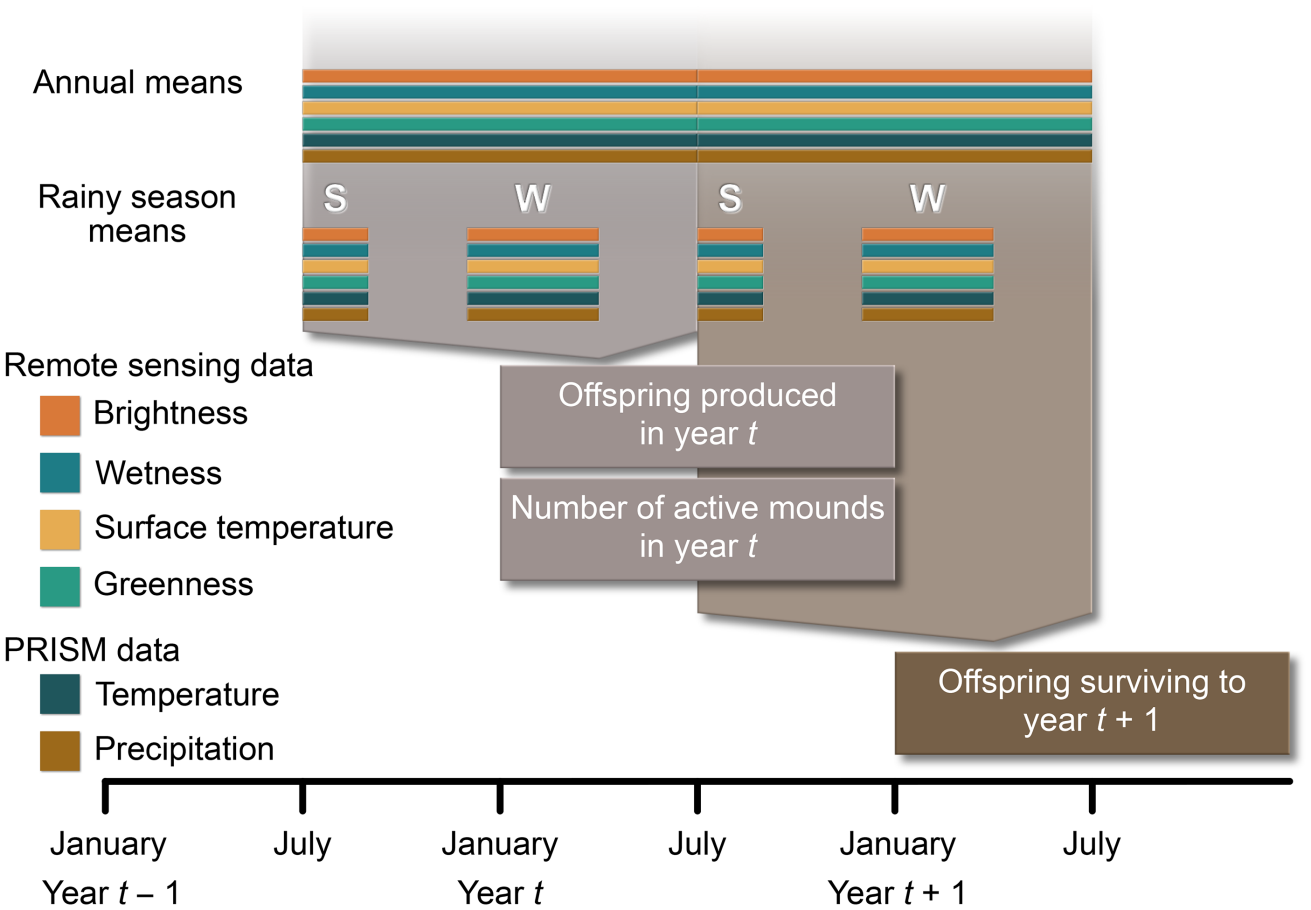
Schematic showing temporal alignments between the predictor and response variables tested in the study. For example: annual means used to predict the number of offspring produced in year *t* were calculated from environmental data collected from July, year *t* − 1 through June, year *t*, whereas winter rainy season means were calculated from data collected from December, year *t* − 1 through March, year *t*. Although not indicated in this figure, PRISM data were only used as predictor variables for population fitness and number of active mounds (i.e., not for measures of individual fitness). Summer and winter rainy season results are indicated by “S” and “W,” respectively.

Using the parent–offspring assignments generated by Waser and Hadfield (2011), we determined how many offspring each female produced in each year (*n* = 476 females) and, for females producing at least one offspring, how many of those offspring survived to age one (i.e., reproductive age; *n* = 282 females). We used the capture data to assign each female to a primary mound location within each year. For each female, we summarized remote sensing values by considering the cell containing her mound location and the eight adjacent cells. Given that each cell is 30 m across, the maximum distance from the center of an individual's home range in the raster to the edge is 63 m. Most banner‐tailed kangaroo rats disperse <50 m over their lifetime (i.e., the distance between their natal and home mounds is <50 m), meaning that their raster‐defined home range likely contains both their natal and reproductive environments (Skvarla et al., 2004). For each year, we calculated mean index and surface temperature values in three ways: (i) season‐equalized 12‐month (i.e., annual) average, wherein the average index values within each meteorological season were averaged to obtain a single annual value for each index; (ii) summary rainy season averages, calculated for July–August; and (iii) winter rainy season averages, calculated for December–March. We applied a 6‐month lag to the environmental data, such that: means for July in year *t* − 1 through June in year *t* were used to predict the number of offspring produced in year *t*; means for July in year *t* through June in year *t* + 1 were used to predict the number of offspring produced in year *t* surviving to year *t* + 1 (Figure 2).

To check for relationships between individual microhabitat conditions and fitness, we conducted a series of Poisson and negative binomial regressions using the *glm.nb* function from the MASS package (Venables & Ripley, 2002) in R. The response variable was number of offspring produced with mean annual values for brightness, greenness, wetness, and surface temperature (K) as predictor variables. We used backward stepwise regression, manually removing one predictor variable at a time and examining model coefficients and AIC values until all predictors were significant (*p* < .05). We compared the final Poisson and negative binomial regressions using a likelihood ratio test, checked the dispersion parameter for each model, and calculated generalized variance inflation factor (GVIF) values for final models with >1 predictor variable retained using the *gvif* function in the glmtoolbox R package to quantify the contribution of collinearity on uncertainty in each model (Hernando Vanegas et al., 2022). We repeated this process for the summer and winter rainy season means and for number of surviving offspring. To visualize the effects of predictor variables in models with multiple retained predictor variables, we used the effect_plot function in the *jtools* package in R, setting the non‐focal predictor variable equal to its mean value.

Because some females were sampled in >1 year, we also constructed negative binomial linear mixed models with female identification number (ID) as a random variable for both number of offspring and number of offspring surviving. These models were built using the *glmmTMB* package in R, and we followed the same backward stepwise regression process as for the models that only included fixed effects (Brooks et al., 2017). For successful mixed models, the final models were compared against the corresponding models lacking random effects with likelihood ratio tests as implemented in the *lrtest* function in R.

#### 2.4.2 Population fitness

We used the capture data to determine the number of adult females alive in each year as well as the total number of offspring produced. From these data, we calculated the average number of offspring produced per female and average number of offspring surviving to age 1 per female. To define the set of cells to be analyzed within each year, we began by identifying active mounds (i.e., mounds where a female was captured) within each year. We defined the total set of active cells as all cells containing an active mound plus the eight cells adjacent to each active cell. For each year, we calculated landscape‐level means for each remote sensing index and the PRISM variables as we did for the individual data (i.e., annually and for the summer and winter rainy seasons) and again applied a 6‐month lag (Figure 2).

We conducted a series of linear regressions to identify relationships between macrohabitat conditions and population‐level fitness by testing each combination of a single environmental predictor variable and response variable separately. Two summer rainy season variables (wetness and brightness) were found to be significant predictors for average number of offspring surviving to age 1 (*p* < .05). Because neither model met the homoskedasticity assumption, we permuted the *y*‐values and calculated model coefficients 1000 times per model to generate permuted *p*‐values.

#### 2.4.3 Population size

Again using capture data, we calculated the number of mounds with resident individuals within each year. We assumed that if a mound was occupied, an experienced surveyor of the site could reasonably identify occupied mounds as active based on signs left by residents (e.g., specific characteristic patterns left by banner‐tailed kangaroo rat locomotion, recently excavated soil at mound entrances). We applied the same predictor variables and statistical approaches as for the population fitness data, using annual and summer and winter rainy season means from July in year *t* − 1 through June in year *t* to predict the number of active mounds in year *t*. After constructing the initial linear regressions, we were left with a single significant predictor variable (mean annual surface temperature; *p* < .05) and again permuted the *y*‐values and calculated model coefficients 1000 times to generate *p*‐values.

To confirm that number of active mounds is a reasonable proxy for population size, we constructed linear models to relate number of active mounds to number of adult females and census population size using the capture data. We also tested for relationships between both number of active mounds and number of adult females and fitness rates (number of offspring and number of surviving offspring per female) to determine whether fitness rates could be the result of density‐dependent population processes. Finally, to account for the effect of population size in year *t* − 1 on population size in year *t*, we repeated our statistical approach using (i) absolute change in population size from year *t* − 1 to year *t* and (ii) proportional change in population size from year *t* − 1 to year *t* as response variables (i.e., $\left(N_{\text{year}\;t}-N_{\text{year}\;t-1}\right)/\left(N_{\text{year}\;t-1}\right)$).

## 3 RESULTS

**FIGURE 3:**
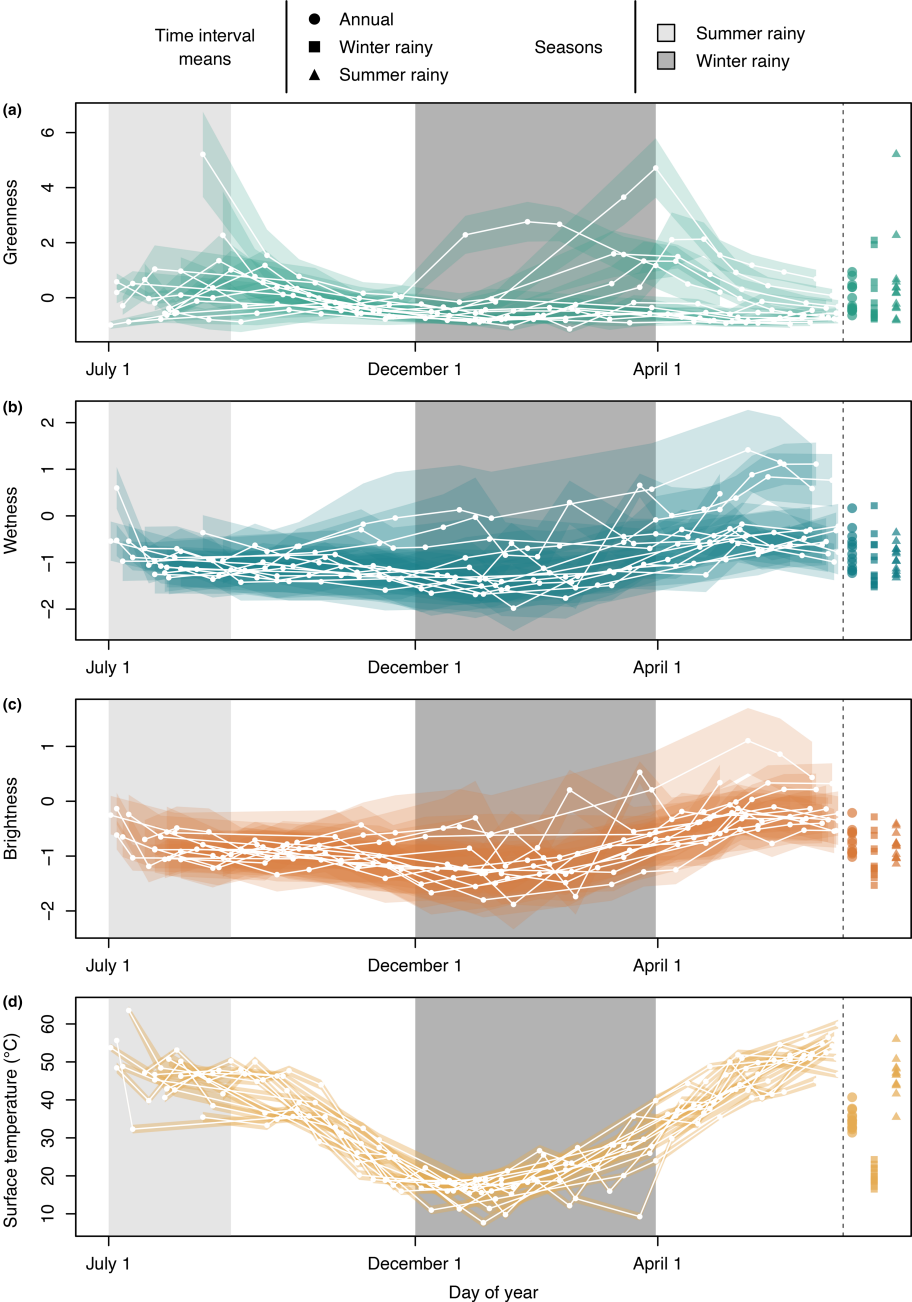
Mean values of Tasseled Cap indices (a–c) and surface temperature (d) across days of the year. Means were calculated using all cells that were occupied in at least 1 year over the course of the study plus all cells directly adjacent to those occupied cells. Note that the *x*‐axes are offset such that the axis begins with July 1 and ends with June 30. White lines connect dates from July 1 in each year through June 30 in the subsequent year. Vertices for shaded polygons encompass one standard deviation around each mean. The points to the right of the dashed line indicate annual and rainy season mean values within each year.

**FIGURE A2:**
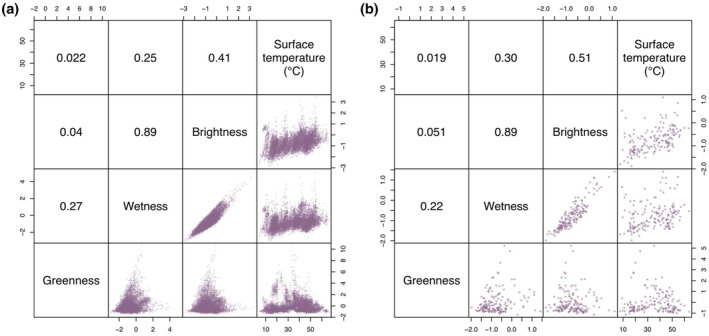
Correlations among remote sensing variables, with Pearson's *r* presented above the diagonals. (a) Points represent 10,000 cells sampled randomly across all time points (i.e., scenes) and all cells active in at least 1 year (*n* = 408 cells). (b) Points represent mean value of active cells within each time point (*n* = 167 scenes). Whereas strong correlation was noted between Tasseled Cap brightness and wetness, the relationships between greenness and these two indices conform to the classic “Tasseled Cap” shape.

We analyzed 167 Landsat 5 TM scenes spanning 13 years (July 1993–June 2005), calculating surface temperature (°C) and scaled and centered Tasseled Cap greenness, wetness, and brightness indices for each. For surface temperature, patterns matched expectations with maximum temperatures observed during June/July and minima during December/January and with little variation across cells analyzed at each time point as indicated by small standard deviations around mean values (Figure 3d). For Tasseled Cap greenness, intra‐annual patterns largely did not follow our expectation of increased values during or after rainy seasons (Figure 3a). For example, higher greenness values were observed for the summer rainy season in only 4 of 13 years analyzed. Brightness and wetness appeared to be strongly correlated with one another, with both indices perhaps decreasing a bit during the cooler months and increasing during the warmer months (Figure 3b,c). After observing the similarities between these two variables, we calculated and confirmed strong correlation between wetness and brightness (Pearson's *r* = .89; Figure A2).

**FIGURE 4:**
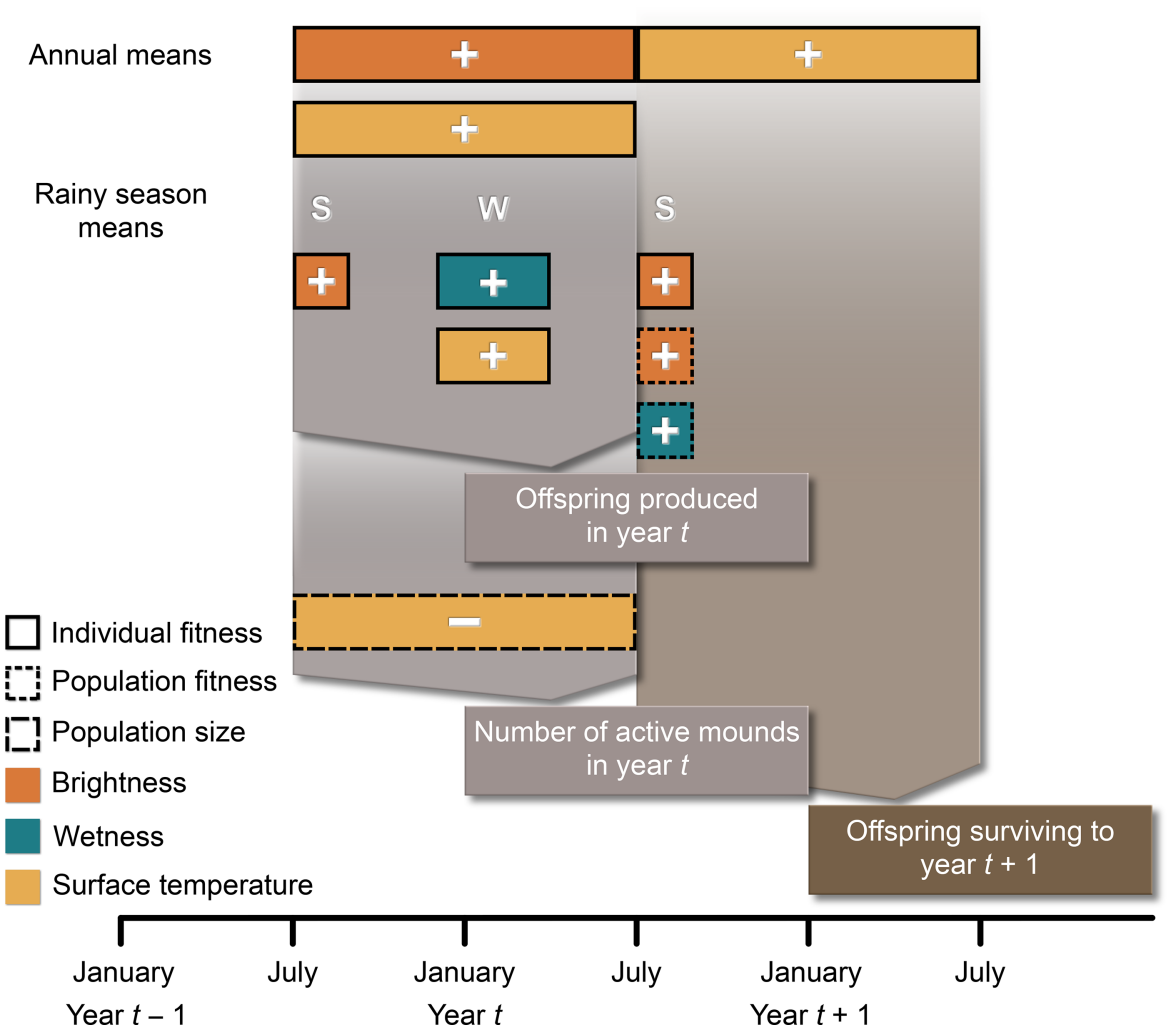
Schematic summarizing the statistically significant relationships identified between environmental variables and fitness or population size. “S” and “W” indicate summer and winter rainy season results, respectively. The sign in each colored polygon indicates the direction of the relationship (i.e., the only negative relationship identified was between mean annual surface temperature and number of active mounds). Polygon color indicates environmental predictor variable with outline pattern indicating the scale at which variables were tested (i.e., individual and population fitness and population size).

**FIGURE 5:**
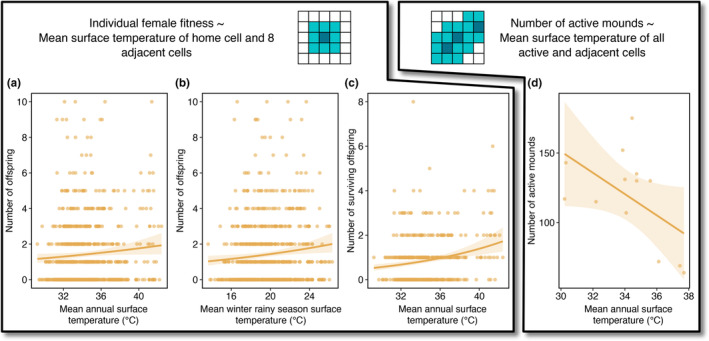
(a–c) Significant positive relationships between surface temperature and measures of individual fitness. Panels a and b present the effects of mean annual and mean winter rainy season surface temperatures, respectively, on number of offspring produced by individual females while setting the non‐focal predictor variable in each negative binomial model equal to its mean value. Panel c presents the final negative binomial model predicting number of surviving offspring with mean annual surface temperature. (d) Linear regression describing negative effect of mean annual surface temperature on population size, as measured by number of active mounds. For all panels, shaded polygons represent 95% confidence intervals. Statistical results for models are presented in Tables 1, 2, 3, 4 and Table A2.

**FIGURE A3:**
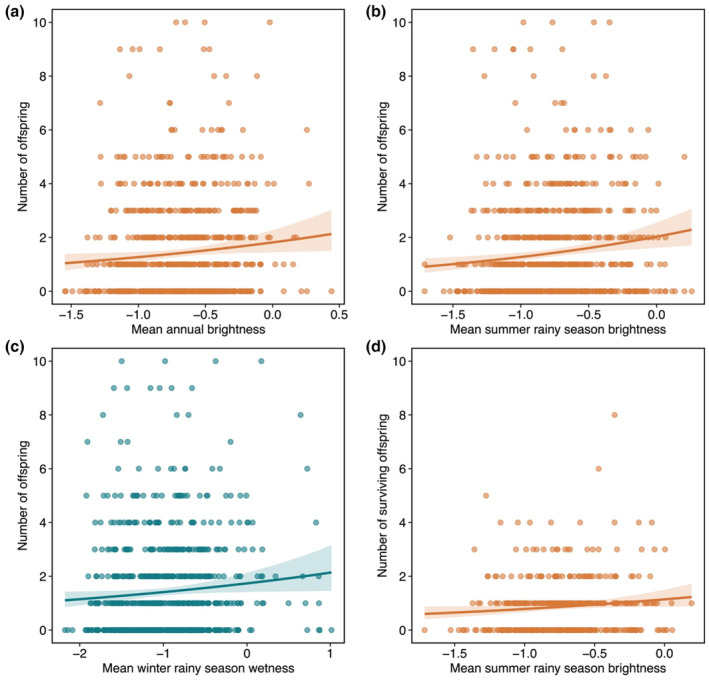
Significant positive relationships between remote sensing measures and individual fitness (a–c, number of offspring; d, number of offspring surviving to age 1). For panels a and c, the effects of each predictor variable were calculated and are presented by setting the non‐focal predictor variable in each negative binomial model equal to its mean value. For all panels, shaded polygons represent 95% confidence intervals. Statistical results for models are presented in Tables 1, A2, and A3.

For individual fitness, we found that mean annual brightness and surface temperature for the area immediately surrounding a female's home location had positive effects on number of offspring produced (Table 1; Figure 4, 5a; Figure A3; see Table A2 for all tested models). Summer rainy season mean brightness and winter rainy season mean wetness and surface temperature also positively affected number of offspring produced (Figure 5b). With respect to the number of offspring surviving to age 1, mean annual surface temperature and mean summer rainy season brightness were positive predictors (Table 1; Figure 5c; see Table A3 for all tested models). For both number of offspring and number of surviving offspring, greenness was not included in any of the final models. For the two individual fitness models with multiple predictor variables retained, GVIF values were close to 1 and therefore did not indicate an outsized contributed of collinearity to model uncertainty (maximum value was 1.05).

**TABLE 1:** Summaries of the best negative binomial models describing individual fitness, with predictor variable values averaged over the time interval indicated.

Fitness measure	Time interval	Variable	Estimate	SE	*z*‐Value	*p*‐Value
Number of offspring (*n* = 476)	Annual	Intercept	−0.7191	0.6523	−1.102	.2703
		Brightness	0.3590	0.1566	2.292	.0219
		Surface temperature	0.0382	0.0187	2.042	.0412
	Summer rainy	Intercept	0.7084	0.1168	6.065	1.32 × 10^−9^
		Brightness	0.4664	0.1430	3.263	.0011
	Winter rainy	Intercept	−0.4845	0.4136	−1.172	.2414
		Wetness	0.2070	0.0973	2.128	.0334
		Surface temperature	0.0524	0.0120	2.628	.0086
Number of surviving offspring (*n* = 282)	Annual	Intercept	−3.2547	0.7081	−4.596	4.3 × 10^−6^
		Surface temperature	0.0896	0.0201	4.457	8.3 × 10^−6^
	Summer rainy	Intercept	0.1346	0.1395	0.965	.3346
		Brightness	0.3752	0.1804	2.080	.0375

Including female ID as a random effect did not affect the results for number of offspring with respect to the identity or significance of retained predictor variables when compared to the negative binomial models that only included fixed effects (tested models presented in Table A4). Likelihood ratio tests comparing mixed‐effects models to models excluding female ID as a random effect were non‐significant. For number of offspring surviving, we could not construct reasonable models that included female ID due to convergence issues. These issues were likely due to the over‐representation of individuals with only 1 year of observations (186 of 282 females were only observed in 1 year). For these reasons, we restrict further consideration and discussion to the results of the models that only include fixed effects for both number of offspring and number of offspring surviving.

**FIGURE A4:**
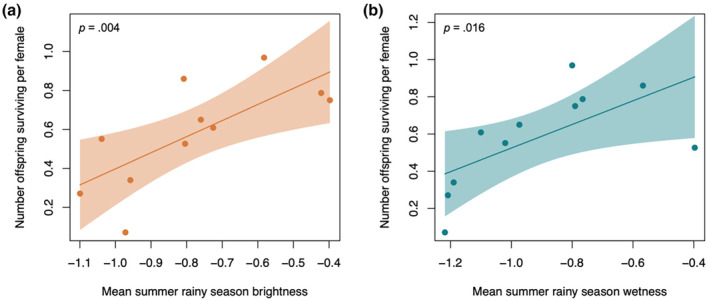
Variables identified as significant predictors of population fitness, specifically the average number of offspring surviving to age 1 per female: mean summer rainy season (a) brightness and (b) wetness. Shaded polygons indicate 95% confidence intervals calculated from the unpermuted linear model. *p*‐Values were calculated from 1000 permutations. Model results for brightness and wetness are presented in Tables 2 and 3, respectively.

There were only two statistically significant relationships linking environmental variables and population fitness: summer rainy season brightness (Table 2) and wetness (Table 3), when averaged across the active landscape, were positively associated with average number of offspring surviving to age 1 per female (Figure A4). Despite summer rainy season wetness positively predicting average number of surviving offspring, total precipitation as modeled by PRISM was not correlated with fitness.

**TABLE 2:** Summary of the first linear regression model describing population fitness and permuted *p*‐values.

Variable	Estimate	SE	*t*‐Value	Permuted *p*‐value
Intercept	1.215	0.210	5.790	<.001
Summer rainy season brightness	0.824	0.262	3.151	.006

*Note*: The response variable is average number of offspring surviving to age 1 per adult female. The adjusted *R* ^2^ for this model was 0.47.

**TABLE 3:** Summary of the second linear regression model describing population fitness and permuted *p*‐values.

Variable	Estimate	SE	*t*‐Value	Permuted *p*‐value
Intercept	1.155	0.237	4.866	.010
Summer rainy season wetness	0.633	0.251	2.518	.010

*Note*: The response variable is average number of offspring surviving to age 1 per adult female. The adjusted *R* ^2^ for this model was 0.35.

**FIGURE A5:**
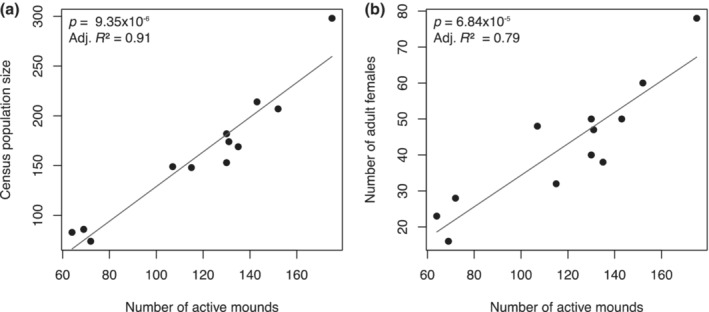
Significant positive relationships between number of active mounds and (a) census population size and (b) number of adult females. Number of active mounds can be reliably ascertained via visual survey of the study site, whereas census population size and number of adult females are both measured via trapping and marking individuals.

**FIGURE A6:**
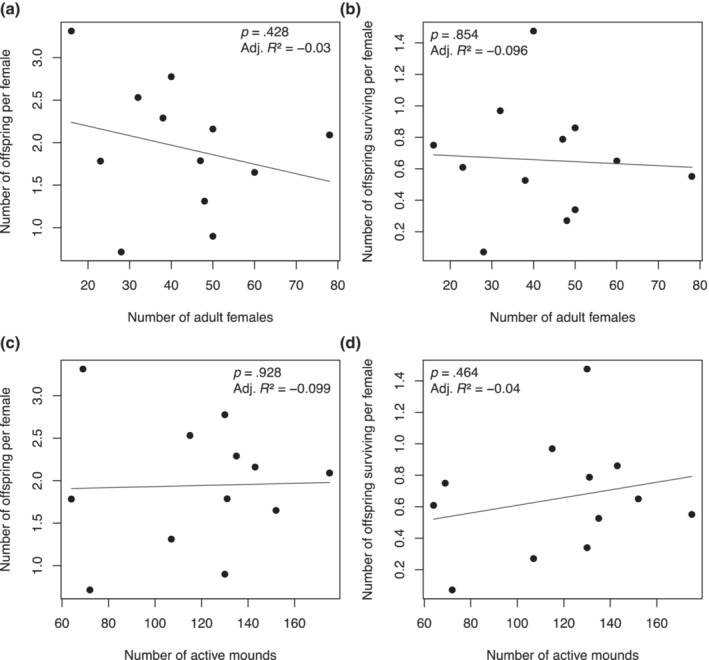
No statistically significant relationships were found between (a,b) number of adult females or (c,d) number of active mounds and average number of offspring per female (a,c) or average number of offspring surviving to age 1 per female (b,d). These patterns suggest a lack of density‐dependent influences on individual fitness for the years included in our study.

With respect to population size, only mean annual surface temperature was a significant predictor variable (Table 4; Figure 5d). The direction of this relationship was negative, unlike the positive relationships described between surface temperature and individual fitness. In comparing number of active mounds against number of adult females and census population sizes, we found significant and strong statistical relationships (Figure A5), suggesting that simply surveying the number of active mounds in an area occupied by banner‐tailed kangaroo rats would produce a close estimate of population size. Neither number of adult females nor number of active mounds were significantly associated with fitness rates (Figure A6), suggesting that fitness is not detectably influenced by population density. We also did not observe any significant relationships between environmental variables and absolute or proportional change in population size from year *t* − 1 to year *t*. Although previous years' population sizes certainly influence contemporary population size, we were not able to capture these effects in our analyses.

**TABLE 4:** Summary of the linear regression model with number of active mounds as the response variable.

Variable	Estimate	SE	*t*‐Value	Permuted *p*‐value
Intercept	380.383	126.632	3.004	.018
Annual surface temperature	−7.644	3.688	−2.073	.018

*Note*: *p*‐Values were derived from permutation tests. The adjusted *R* ^2^ for this model was 0.22.

## 4 DISCUSSION

For two variables—Tasseled Cap brightness and wetness—our results matched our expectations that were based on previously published relationships between kangaroo rat demographic measures and environmental conditions. In each individual‐level model where brightness was retained as a significant predictor and at the population level, brightness positively affected fitness. This is consistent with previous studies that explicitly tested the relationship between habitat openness (i.e., plant density or shrub cover) and kangaroo rat abundance (Bowers et al., 1987; Waser & Ayers, 2003). However, these studies primarily focused on the effect of shrub density on kangaroo rat populations, whereas the majority of plants at our study site are grasses. Therefore, brightness as measured in our study may be providing a summary of favorable conditions distinct from what was explicitly tested in previous studies of habitat openness and kangaroo rat abundance. Mean winter and summer rainy season wetness values were also positively associated with individual and population fitness, respectively. This mirrors results of previous studies that have demonstrated a positive relationship between precipitation and rodent abundances in dry environments (Cárdenas et al., 2021), although mechanistic links between precipitation and rodent abundances are often complex (Ernest et al., 2000; Thibault et al., 2010; Thibault & Brown, 2008). Positive effects of precipitation on kangaroo rat survival or abundance could be mediated via decreased water stress on individuals or through increased availability of food resources that rely on rainy season precipitation to produce seeds. However, values for wetness and brightness were strongly correlated in our dataset (Pearson's *r* = .89; Figure 3b,c; Figure A2), making it difficult to definitively interpret changes in either index. This is likely due to the high ratio of bare soil: vegetation cover at our site, with little variation across the cells being compared (Crist, 1985). The strength of this correlation does vary across the year (summer rainy season Pearson's *r* = .57; winter rainy season Pearson's *r* = .92), indicating that these two indices likely capture distinct soil characteristics, but we cannot explicitly define those characteristics without ground‐truthed data.

Across all time intervals and scales, Tasseled Cap greenness was never retained as a significant predictor of fitness or population size. We expected greenness to increase during or immediately following the rainy seasons in each year, and this appears to have been the case for some years but not all (Figure 3). The uninformative nature of this particular index for our study site is likely related to semiarid shrub and grassland characteristics. In such ecosystems, spatial patterns of vegetative land cover are highly heterogeneous with respect to both plant community composition and density (Huenneke et al., 2001) and typically comprise dormant (i.e., non‐photosynthetic) vegetative cover for large portions of the year (Browning et al., 2017; Okin, 2010; Yang & Guo, 2014). Regardless of season, areas with sparse vegetative cover may not reach greenness thresholds required for detection of vegetation in satellite data (Peng et al., 2021). In other words, the low density of green vegetation at our study site may not be sufficient to prompt an increase in Tasseled Cap greenness values on a per‐cell basis, even when the plant community has reached maximum greenness. Further investigation would require either higher resolution data than is publicly available (e.g., Bankert et al., 2021; Browning et al., 2019) or ground‐truthed data to calibrate conversions of spectral data to per‐pixel vegetation fractions (Smith et al., 1990) on a temporal scale capable of capturing the often rapid changes in photosynthetic activity observed in desert plants (Reed et al., 1994). Without such information from the focal system, it may not possible to reliably ascertain aspects of shrub or grassland phenology using multispectral data alone (Allnutt et al., 2002).

Whereas the Tasseled Cap indices may require additional data to contextualize their values for a specific location, the Landsat surface temperature band provides a direct measure of a simple physical characteristic. At the individual level, the positive relationships we identified between surface temperature and individual fitness appear to directly contradict other findings in this species (Moses et al., 2012). However, when we analyzed the effect of surface temperature on population size, the direction of this relationship matched previous results describing negative effects of increased temperature on kangaroo rat survival. The apparent mismatch between these two sets of results could be mediated by decoupled processes acting over distinct time frames to increase both individual fitness and subsequent overall mortality in the population. Specifically, the positive effect of surface temperature on individual fitness is partially driven by higher winter temperatures (as was found for the number of offspring response variable), and warmer winters correspond to lower thermoregulatory costs for kangaroo rats (Edelman, 2011; Hinds & MacMillen, 1985). These reduced costs could help the kangaroo rats' seed caches to last longer, allowing females to produce greater numbers of litters in a single season. Whereas higher winter temperatures may correspond to greater numbers of offspring produced, higher summer temperatures may lead to higher rates of mortality. Although we did not detect a significant relationship between summer rainy season surface temperature and population size, hotter summers could perhaps decrease plant productivity, leading kangaroo rats to quickly exhaust their seed caches and spend more time gathering food at night, thereby also increasing their risk of predation. Additional environmental data (e.g., accurate measurements of plant community composition, abundance, and phenology) could provide greater context for interpreting the influence of surface temperature on population dynamics, but satellite‐measured surface temperature alone may remain a critical and accessible measure of habitat suitability or population dynamics for many species as climate change progresses, including species of conservation concern and pest species (Albright et al., 2011; Bateman et al., 2023; Blum et al., 2015; Geppert et al., 2023; Moses et al., 2012; Shimada et al., 2021).

Although four of our final models included surface temperature as a predictor variable, PRISM temperature estimates were never retained as significant predictors of fitness or population size, nor were PRISM precipitation estimates. One possible explanation is that PRISM estimates may not closely approximate the true values for our study site, which covers roughly 6% of a single PRISM grid cell. PRISM models account for elevation and topography, but precipitation is highly spatially variable in the Chihuahuan Desert, even over short distances (Petrie et al., 2014), making it difficult to assess the accuracy of PRISM estimates over very small areas. Additionally, large precipitation events can contribute the majority of annual rainfall in wet years (Petrie et al., 2014), and extreme weather events could influence kangaroo rat fitness or survival more strongly than the seasonal or annual averages (e.g., due to food resource spoilage (Valone et al., 1995)) we included in our analyses. Spatial variability in air temperatures may also impede detection of relationships between the modeled PRISM temperatures, but a more likely explanation is that surface temperature values are simply more representative of the environment kangaroo rats experience than air temperature estimates, further highlighting the utility of remotely sensed surface temperature measurements in this and similar habitats.

For all of the environmental variables we tested, we also checked whether these variables were predictive of either absolute or proportional change in population size from 1 year to the next. Population size in the preceding year certainly influences contemporary population size, but we did not detect any relationships between environmental variables and either measure of change in population size. It may be that surface temperature—the only variable significantly associated with population size—is also correlated with some unmeasured aspect of the environment that limits population carrying capacity rather than rate of change in population size. We did find that number of active mounds is reliably predictive of population size, as has been previously described for this population over a different set of sampling years (Cross & Waser, 2000). Although visual surveys of the site would not provide information on individual fitness, they could provide close estimates of population size with far less effort than extensive trapping schemes. Future studies of this or other *D. spectabilis* populations could rely on more extensive ground‐truthed remote sensing data and active mound surveys to gain additional insights into drivers of population size while minimizing the number of person hours required to collect data.

## 5 CONCLUSIONS

Through our analysis of remote sensing and modeled climate data, we were able to identify potential ecological drivers of fitness and population size. Although most of our tested variables (i.e., the Tasseled Cap indices) will require pairing with ground‐truthed data from the site to confirm, our results and conservative interpretations were consistent with previous findings from our focal population and other systems. The contrasting results for surface temperature across sampling scales demonstrate that, while conducting relatively lower effort mound surveys likely captures demographic trends well enough to identify abiotic determinants of population size, the additional resolution provided by linking parents and offspring via genetic sampling allows for the detection of counterintuitive relationships that could influence long‐term population stability.

## AUTHOR CONTRIBUTIONS

**Avril M. Harder:** Conceptualization (equal); data curation (lead); formal analysis (lead); methodology (lead); visualization (lead); writing – original draft (lead); writing – review and editing (equal). **Mekala Sundaram:** Methodology (equal); writing – review and editing (equal). **Lana L. Narine:** Methodology (equal); writing – review and editing (equal). **Janna R. Willoughby:** Conceptualization (equal); writing – review and editing (equal).

## Data Availability

Code for all analyses is available at https://github.com/avril‐m‐harder/krat_remote_sensing_scripts. All final datasets are available in Dryad (doi: 10.5061/dryad.9p8cz8wnf).

## APPENDIX A

**TABLE A1:** Metadata for final list of Landsat 5 TM Collection 2 Level 2 scenes (path 35, row 38) used in analyses (*n* = 167).

Product ID	Scene ID	Date			Time		
		Year	Month	Day	Hour	Minute	Second
LT05_L2SP_035038_19930104_20200914_02_T1	LT50350381993004AAA04	1993	1	4	10	13	33
LT05_L2SP_035038_19930309_20200914_02_T1	LT50350381993068AAA04	1993	3	9	10	14	12
LT05_L2SP_035038_19930325_20200914_02_T1	LT50350381993084AAA04	1993	3	25	10	14	12
LT05_L2SP_035038_19930410_20200914_02_T1	LT50350381993100AAA04	1993	4	10	10	14	20
LT05_L2SP_035038_19930512_20200914_02_T1	LT50350381993132AAA04	1993	5	12	10	14	31
LT05_L2SP_035038_19930528_20200914_02_T1	LT50350381993148AAA04	1993	5	28	10	14	34
LT05_L2SP_035038_19930613_20200914_02_T1	LT50350381993164AAA04	1993	6	13	10	14	34
LT05_L2SP_035038_19930629_20200914_02_T1	LT50350381993180AAA04	1993	6	29	10	14	27
LT05_L2SP_035038_19930731_20200913_02_T1	LT50350381993212AAA04	1993	7	31	10	14	26
LT05_L2SP_035038_19930917_20200913_02_T1	LT50350381993260XXX03	1993	9	17	10	14	22
LT05_L2SP_035038_19931003_20200913_02_T1	LT50350381993276AAA03	1993	10	3	10	14	18
LT05_L2SP_035038_19931104_20200913_02_T1	LT50350381993308AAA04	1993	11	4	10	14	6
LT05_L2SP_035038_19931120_20200913_02_T1	LT50350381993324XXX04	1993	11	20	10	13	58
LT05_L2SP_035038_19931206_20200913_02_T1	LT50350381993340XXX05	1993	12	6	10	13	55
LT05_L2SP_035038_19940107_20200913_02_T1	LT50350381994007XXX01	1994	1	7	10	13	33
LT05_L2SP_035038_19940328_20200913_02_T1	LT50350381994087XXX02	1994	3	28	10	12	27
LT05_L2SP_035038_19940413_20200913_02_T1	LT50350381994103XXX02	1994	4	13	10	12	7
LT05_L2SP_035038_19940429_20200913_02_T1	LT50350381994119XXX02	1994	4	29	10	11	51
LT05_L2SP_035038_19940515_20200913_02_T1	LT50350381994135XXX02	1994	5	15	10	11	33
LT05_L2SP_035038_19940616_20200913_02_T1	LT50350381994167XXX02	1994	6	16	10	10	56
LT05_L2SP_035038_19940702_20200913_02_T1	LT50350381994183XXX02	1994	7	2	10	10	32
LT05_L2SP_035038_19940803_20200913_02_T1	LT50350381994215XXX02	1994	8	3	10	9	38
LT05_L2SP_035038_19941022_20200912_02_T1	LT50350381994295XXX02	1994	10	22	10	7	28
LT05_L2SP_035038_19941209_20200912_02_T1	LT50350381994343XXX02	1994	12	9	10	5	52
LT05_L2SP_035038_19950110_20200912_02_T1	LT50350381995010XXX01	1995	1	10	10	4	47
LT05_L2SP_035038_19950315_20200912_02_T1	LT50350381995074AAA01	1995	3	15	10	2	15
LT05_L2SP_035038_19950331_20200912_02_T1	LT50350381995090XXX01	1995	3	31	10	1	36
LT05_L2SP_035038_19950502_20200912_02_T1	LT50350381995122XXX01	1995	5	2	10	0	13
LT05_L2SP_035038_19950518_20200912_02_T1	LT50350381995138XXX02	1995	5	18	9	59	30
LT05_L2SP_035038_19950603_20200913_02_T1	LT50350381995154XXX01	1995	6	3	9	58	45
LT05_L2SP_035038_19950619_20200913_02_T1	LT50350381995170XXX03	1995	6	19	9	58	2
LT05_L2SP_035038_19950705_20200912_02_T1	LT50350381995186XXX02	1995	7	5	9	57	19
LT05_L2SP_035038_19950721_20200912_02_T1	LT50350381995202XXX02	1995	7	21	9	56	35
LT05_L2SP_035038_19950806_20200912_02_T1	LT50350381995218AAA02	1995	8	6	9	55	51
LT05_L2SP_035038_19950822_20200912_02_T1	LT50350381995234XXX02	1995	8	22	9	55	7
LT05_L2SP_035038_19950923_20200912_02_T1	LT50350381995266XXX02	1995	9	23	9	53	34
LT05_L2SP_035038_19951009_20200912_02_T1	LT50350381995282XXX03	1995	10	9	9	52	44
LT05_L2SP_035038_19951025_20200912_02_T1	LT50350381995298AAA01	1995	10	25	9	51	50
LT05_L2SP_035038_19951110_20200912_02_T1	LT50350381995314XXX00	1995	11	10	9	51	30
LT05_L2SP_035038_19951212_20200911_02_T1	LT50350381995346XXX01	1995	12	12	9	52	54
LT05_L2SP_035038_19960113_20200911_02_T1	LT50350381996013XXX01	1996	1	13	9	55	13
LT05_L2SP_035038_19960129_20200912_02_T1	LT50350381996029AAA01	1996	1	29	9	56	20
LT05_L2SP_035038_19960214_20200912_02_T1	LT50350381996045XXX01	1996	2	14	9	57	26
LT05_L2SP_035038_19960317_20200911_02_T1	LT50350381996077XXX01	1996	3	17	9	59	34
LT05_L2SP_035038_19960418_20200911_02_T1	LT50350381996109AAA02	1996	4	18	10	1	34
LT05_L2SP_035038_19960504_20200911_02_T1	LT50350381996125AAA01	1996	5	4	10	2	31
LT05_L2SP_035038_19960520_20200911_02_T1	LT50350381996141XXX02	1996	5	20	10	3	26
LT05_L2SP_035038_19960605_20200911_02_T1	LT50350381996157XXX02	1996	6	5	10	4	20
LT05_L2SP_035038_19960707_20200911_02_T1	LT50350381996189XXX03	1996	7	7	10	6	2
LT05_L2SP_035038_19960723_20200911_02_T1	LT50350381996205AAA02	1996	7	23	10	6	53
LT05_L2SP_035038_19961011_20200911_02_T1	LT50350381996285XXX02	1996	10	11	10	11	10
LT05_L2SP_035038_19961112_20200911_02_T1	LT50350381996317XXX02	1996	11	12	10	12	43
LT05_L2SP_035038_19961214_20200910_02_T1	LT50350381996349XXX02	1996	12	14	10	14	14
LT05_L2SP_035038_19961230_20200910_02_T1	LT50350381996365AAA02	1996	12	30	10	15	0
LT05_L2SP_035038_19970115_20200910_02_T1	LT50350381997015XXX02	1997	1	15	10	15	45
LT05_L2SP_035038_19970131_20200910_02_T1	LT50350381997031XXX01	1997	1	31	10	16	28
LT05_L2SP_035038_19970216_20200910_02_T1	LT50350381997047XXX02	1997	2	16	10	17	9
LT05_L2SP_035038_19970320_20200910_02_T1	LT50350381997079AAA02	1997	3	20	10	18	26
LT05_L2SP_035038_19970421_20200910_02_T1	LT50350381997111XXX02	1997	4	21	10	19	35
LT05_L2SP_035038_19970507_20200910_02_T1	LT50350381997127XXX02	1997	5	7	10	20	8
LT05_L2SP_035038_19970523_20200910_02_T1	LT50350381997143XXX02	1997	5	23	10	20	42
LT05_L2SP_035038_19970624_20200910_02_T1	LT50350381997175AAA02	1997	6	24	10	21	49
LT05_L2SP_035038_19970827_20200909_02_T1	LT50350381997239AAA02	1997	8	27	10	23	55
LT05_L2SP_035038_19970912_20200909_02_T1	LT50350381997255XXX02	1997	9	12	10	24	22
LT05_L2SP_035038_19970928_20200909_02_T1	LT50350381997271AAA02	1997	9	28	10	24	50
LT05_L2SP_035038_19971014_20200910_02_T1	LT50350381997287XXX02	1997	10	14	10	25	16
LT05_L2SP_035038_19971217_20200909_02_T1	LT50350381997351AAA01	1997	12	17	10	26	52
LT05_L2SP_035038_19980102_20200909_02_T1	LT50350381998002AAA02	1998	1	2	10	27	15
LT05_L2SP_035038_19980118_20200909_02_T1	LT50350381998018AAA02	1998	1	18	10	27	35
LT05_L2SP_035038_19980203_20200909_02_T1	LT50350381998034AAA01	1998	2	3	10	27	57
LT05_L2SP_035038_19980219_20200909_02_T1	LT50350381998050AAA02	1998	2	19	10	28	16
LT05_L2SP_035038_19980323_20200909_02_T1	LT50350381998082XXX01	1998	3	23	10	28	45
LT05_L2SP_035038_19980408_20200909_02_T1	LT50350381998098XXX01	1998	4	8	10	28	57
LT05_L2SP_035038_19980424_20200909_02_T1	LT50350381998114XXX02	1998	4	24	10	29	9
LT05_L2SP_035038_19980510_20200909_02_T1	LT50350381998130AAA02	1998	5	10	10	29	24
LT05_L2SP_035038_19980526_20200909_02_T1	LT50350381998146XXX02	1998	5	26	10	29	40
LT05_L2SP_035038_19980627_20200909_02_T1	LT50350381998178XXX02	1998	6	27	10	30	4
LT05_L2SP_035038_19980729_20200909_02_T1	LT50350381998210XXX02	1998	7	29	10	30	23
LT05_L2SP_035038_19980814_20200909_02_T1	LT50350381998226XXX02	1998	8	14	10	30	30
LT05_L2SP_035038_19981102_20200908_02_T1	LT50350381998306XXX01	1998	11	2	10	30	56
LT05_L2SP_035038_19981118_20200908_02_T1	LT50350381998322XXX01	1998	11	18	10	31	4
LT05_L2SP_035038_19981204_20200908_02_T1	LT50350381998338XXX02	1998	12	4	10	30	59
LT05_L2SP_035038_19981220_20200908_02_T1	LT50350381998354AAA02	1998	12	20	10	31	4
LT05_L2SP_035038_19990105_20200908_02_T1	LT50350381999005XXX01	1999	1	5	10	31	6
LT05_L2SP_035038_19990206_20200908_02_T1	LT50350381999037XXX01	1999	2	6	10	31	12
LT05_L2SP_035038_19990427_20200908_02_T1	LT50350381999117AAA02	1999	4	27	10	30	45
LT05_L2SP_035038_19990529_20200908_02_T1	LT50350381999149AAA01	1999	5	29	10	30	11
LT05_L2SP_035038_19990630_20200908_02_T1	LT50350381999181AAA02	1999	6	30	10	29	50
LT05_L2SP_035038_19990817_20200907_02_T1	LT50350381999229XXX01	1999	8	17	10	29	36
LT05_L2SP_035038_19990918_20200907_02_T1	LT50350381999261XXX01	1999	9	18	10	28	41
LT05_L2SP_035038_19991004_20200907_02_T1	LT50350381999277AAA02	1999	10	4	10	28	38
LT05_L2SP_035038_19991105_20200907_02_T1	LT50350381999309XXX02	1999	11	5	10	28	5
LT05_L2SP_035038_19991121_20200907_02_T1	LT50350381999325XXX03	1999	11	21	10	27	28
LT05_L2SP_035038_19991223_20200907_02_T1	LT50350381999357XXX02	1999	12	23	10	27	7
LT05_L2SP_035038_20000108_20200907_02_T1	LT50350382000008XXX02	2000	1	8	10	27	4
LT05_L2SP_035038_20000328_20200907_02_T1	LT50350382000088XXX02	2000	3	28	10	26	23
LT05_L2SP_035038_20000515_20200907_02_T1	LT50350382000136XXX00	2000	5	15	10	27	49
LT05_L2SP_035038_20000531_20200907_02_T1	LT50350382000152XXX02	2000	5	31	10	28	5
LT05_L2SP_035038_20000616_20200907_02_T1	LT50350382000168XXX02	2000	6	16	10	28	26
LT05_L2SP_035038_20000718_20200906_02_T1	LT50350382000200XXX02	2000	7	18	10	29	6
LT05_L2SP_035038_20000803_20200906_02_T1	LT50350382000216XXX02	2000	8	3	10	29	18
LT05_L2SP_035038_20000819_20200907_02_T1	LT50350382000232XXX02	2000	8	19	10	29	45
LT05_L2SP_035038_20000904_20200906_02_T1	LT50350382000248XXX02	2000	9	4	10	30	8
LT05_L2SP_035038_20001123_20200906_02_T1	LT50350382000328XXX02	2000	11	23	10	31	12
LT05_L2SP_035038_20001225_20200906_02_T1	LT50350382000360XXX03	2000	12	25	10	31	43
LT05_L2SP_035038_20010126_20200906_02_T1	LT50350382001026XXX02	2001	1	26	10	31	57
LT05_L2SP_035038_20010211_20200906_02_T1	LT50350382001042XXX02	2001	2	11	10	32	3
LT05_L2SP_035038_20010315_20200906_02_T1	LT50350382001074XXX02	2001	3	15	10	32	10
LT05_L2SP_035038_20010331_20200906_02_T1	LT50350382001090AAA02	2001	3	31	10	32	9
LT05_L2SP_035038_20010416_20200906_02_T1	LT50350382001106XXX02	2001	4	16	10	32	1
LT05_L2SP_035038_20010502_20200906_02_T1	LT50350382001122XXX02	2001	5	2	10	32	14
LT05_L2SP_035038_20010806_20200906_02_T1	LT50350382001218LGS01	2001	8	6	10	32	22
LT05_L2SP_035038_20010822_20200905_02_T1	LT50350382001234LGS01	2001	8	22	10	32	18
LT05_L2SP_035038_20010907_20200906_02_T1	LT50350382001250LGS01	2001	9	7	10	32	12
LT05_L2SP_035038_20010923_20200905_02_T1	LT50350382001266LGS01	2001	9	23	10	32	5
LT05_L2SP_035038_20011025_20200905_02_T1	LT50350382001298LGS01	2001	10	25	10	31	50
LT05_L2SP_035038_20011228_20200905_02_T1	LT50350382001362LGS01	2001	12	28	10	31	18
LT05_L2SP_035038_20020113_20200905_02_T1	LT50350382002013EDC01	2002	1	13	10	31	6
LT05_L2SP_035038_20020214_20200905_02_T1	LT50350382002045LGS01	2002	2	14	10	30	36
LT05_L2SP_035038_20020302_20200905_02_T1	LT50350382002061LGS01	2002	3	2	10	30	17
LT05_L2SP_035038_20020521_20200905_02_T1	LT50350382002141LGS01	2002	5	21	10	28	49
LT05_L2SP_035038_20020606_20200905_02_T1	LT50350382002157LGS01	2002	6	6	10	28	32
LT05_L2SP_035038_20020622_20200905_02_T1	LT50350382002173LGS03	2002	6	22	10	28	5
LT05_L2SP_035038_20020724_20200905_02_T1	LT50350382002205LGS01	2002	7	24	10	27	23
LT05_L2SP_035038_20020825_20200905_02_T1	LT50350382002237LGS01	2002	8	25	10	26	22
LT05_L2SP_035038_20020926_20200905_02_T1	LT50350382002269LGS01	2002	9	26	10	25	37
LT05_L2SP_035038_20021113_20200905_02_T1	LT50350382002317EDC01	2002	11	13	10	24	0
LT05_L2SP_035038_20021231_20200905_02_T1	LT50350382002365LGS01	2002	12	31	10	24	6
LT05_L2SP_035038_20030116_20200905_02_T1	LT50350382003016LGS01	2003	1	16	10	24	27
LT05_L2SP_035038_20030201_20200905_02_T1	LT50350382003032LGS01	2003	2	1	10	24	48
LT05_L2SP_035038_20030217_20200904_02_T1	LT50350382003048LGS01	2003	2	17	10	25	8
LT05_L2SP_035038_20030406_20200904_02_T1	LT50350382003096LGS01	2003	4	6	10	26	24
LT05_L2SP_035038_20030422_20200905_02_T1	LT50350382003112LGS01	2003	4	22	10	26	48
LT05_L2SP_035038_20030508_20200905_02_T1	LT50350382003128LGS01	2003	5	8	10	27	8
LT05_L2SP_035038_20030625_20200905_02_T1	LT50350382003176LGS01	2003	6	25	10	28	5
LT05_L2SP_035038_20030711_20200905_02_T1	LT50350382003192PAC02	2003	7	11	10	28	23
LT05_L2SP_035038_20030727_20200904_02_T1	LT50350382003208PAC02	2003	7	27	10	28	41
LT05_L2SP_035038_20030913_20200904_02_T1	LT50350382003256PAC02	2003	9	13	10	29	32
LT05_L2SP_035038_20030929_20200904_02_T1	LT50350382003272PAC02	2003	9	29	10	29	43
LT05_L2SP_035038_20031015_20200904_02_T1	LT50350382003288PAC02	2003	10	15	10	29	56
LT05_L2SP_035038_20031116_20200904_02_T1	LT50350382003320LGS01	2003	11	16	10	30	21
LT05_L2SP_035038_20031218_20200904_02_T1	LT50350382003352PAC02	2003	12	18	10	30	40
LT05_L2SP_035038_20040103_20200904_02_T1	LT50350382004003LGS01	2004	1	3	10	30	48
LT05_L2SP_035038_20040119_20200904_02_T1	LT50350382004019LGS01	2004	1	19	10	30	50
LT05_L2SP_035038_20040220_20200903_02_T1	LT50350382004051LGS01	2004	2	20	10	30	59
LT05_L2SP_035038_20040307_20200903_02_T1	LT50350382004067PAC02	2004	3	7	10	31	4
LT05_L2SP_035038_20040424_20200903_02_T1	LT50350382004115PAC02	2004	4	24	10	32	0
LT05_L2SP_035038_20040510_20200903_02_T1	LT50350382004131PAC04	2004	5	10	10	32	28
LT05_L2SP_035038_20040611_20200903_02_T1	LT50350382004163PAC02	2004	6	11	10	33	22
LT05_L2SP_035038_20040627_20200903_02_T1	LT50350382004179EDC00	2004	6	27	10	33	51
LT05_L2SP_035038_20040729_20200903_02_T1	LT50350382004211EDC00	2004	7	29	10	34	41
LT05_L2SP_035038_20040830_20200903_02_T1	LT50350382004243EDC00	2004	8	30	10	35	28
LT05_L2SP_035038_20040915_20200903_02_T1	LT50350382004259EDC00	2004	9	15	10	35	53
LT05_L2SP_035038_20041102_20200903_02_T1	LT50350382004307EDC00	2004	11	2	10	36	55
LT05_L2SP_035038_20041220_20200902_02_T1	LT50350382004355EDC00	2004	12	20	10	37	50
LT05_L2SP_035038_20050310_20200902_02_T1	LT50350382005069EDC00	2005	3	10	10	38	57
LT05_L2SP_035038_20050411_20200902_02_T1	LT50350382005101EDC00	2005	4	11	10	39	9
LT05_L2SP_035038_20050427_20200902_02_T1	LT50350382005117EDC00	2005	4	27	10	39	13
LT05_L2SP_035038_20050513_20200902_02_T1	LT50350382005133EDC00	2005	5	13	10	39	19
LT05_L2SP_035038_20050614_20200902_02_T1	LT50350382005165EDC00	2005	6	14	10	39	35
LT05_L2SP_035038_20050630_20200902_02_T1	LT50350382005181EDC00	2005	6	30	10	39	39
LT05_L2SP_035038_20050716_20200902_02_T1	LT50350382005197EDC00	2005	7	16	10	39	49
LT05_L2SP_035038_20050801_20200902_02_T1	LT50350382005213EDC00	2005	8	1	10	39	59
LT05_L2SP_035038_20050918_20200901_02_T1	LT50350382005261PAC01	2005	9	18	10	40	10
LT05_L2SP_035038_20051020_20200901_02_T1	LT50350382005293PAC01	2005	10	20	10	40	5
LT05_L2SP_035038_20051105_20200901_02_T1	LT50350382005309PAC01	2005	11	5	10	40	12
LT05_L2SP_035038_20051121_20201008_02_T1	LT50350382005325PAC01	2005	11	21	10	40	36

*Note*: Time is provided in Mountain Standard Time (local to the study site).

**TABLE A2:** Summary of all models tested using backward stepwise regression for each regression type (P = Poisson, NB = negative binomial) and time interval.

Time interval	Regression type	Model	AIC	Residual df	Dispersion value	Log‐likelihood
Annual	P	I + greenness + brightness + wetness + surface temperature	2980.5	786	2.40	−1485.26
Annual	P	I + brightness + wetness + surface temperature	2978.5	787	2.39	−1485.26
Annual	P	I + brightness + surface temperature	2976.5	788	2.39	−1485.26
Annual	NB	I + greenness + brightness + wetness + surface temperature	2634.5	786	0.96	−1311.24
Annual	NB	I + greenness + brightness + surface temperature	2632.5	787	0.96	−1311.25
**Annual**	**NB**	**I + brightness + surface temperature**	**2630.5**	**788**	**0.96**	**−1311.27**
Summer rainy	P	I + greenness + brightness + wetness + surface temperature	2968.5	786	2.40	−1479.28
Summer rainy	P	I + brightness + wetness + surface temperature	2966.7	787	2.40	−1479.35
Summer rainy	NB	I + greenness + brightness + wetness + surface temperature	2630.0	786	0.98	−1309.00
Summer rainy	NB	I + brightness + wetness + surface temperature	2628.0	787	0.98	−1309.02
Summer rainy	NB	I + brightness + surface temperature	2627.6	788	0.98	−1309.80
**Summer rainy**	**NB**	**I + brightness**	**2626.7**	**789**	**0.97**	**−1310.36**
Winter rainy	P	I + greenness + brightness + wetness + surface temperature	2840.1	748	2.46	−1415.06
Winter rainy	P	I + brightness + wetness + surface temperature	2838.2	749	2.46	−1415.12
Winter rainy	P	I + wetness + surface temperature	2839.2	750	2.46	−1416.61
Winter rainy	NB	I + greenness + brightness + wetness + surface temperature	2488.3	748	0.96	−1238.14
Winter rainy	NB	I + brightness + wetness + surface temperature	2486.4	749	0.96	−1238.22
**Winter rainy**	**NB**	**I + wetness + surface temperature**	**2486.2**	**750**	**0.95**	**−1239.09**

*Note*: For all models in the table, the response variable was individual fitness as measured by number of offspring produced. The final model selected for each time interval is bolded. In all cases, negative binomial was a better fit than Poisson with the same predictor variables, according to a likelihood ratio test for each final model.

**TABLE A3:** Summary of all models tested using backward stepwise regression for each regression type (P = Poisson, NB = negative binomial) and time interval.

Time interval	Regression type	Model	AIC	Residual df	Dispersion value	Log‐likelihood
Annual	P	I + greenness + brightness + wetness + surface temperature	1046.4	410	1.23	−518.20
Annual	P	I + greenness + wetness + surface temperature	1044.6	411	1.23	−518.29
Annual	P	I + wetness + surface temperature	1043.6	412	1.24	−518.78
Annual	P	I + surface temperature	1043.1	413	1.25	−519.53
Annual	NB	I + greenness + brightness + wetness + surface temperature	1042.5	410	1.06	−515.24
Annual	NB	I + greenness + wetness + surface temperature	1040.6	411	1.06	−515.31
Annual	NB	I + wetness + surface temperature	1039.5	412	1.06	−515.76
**Annual**	**NB**	**I + surface temperature**	**1038.8**	**413**	**1.07**	**−516.39**
Summer rainy	P	I + greenness + brightness + wetness + surface temperature	997.4	384	1.20	−493.71
Summer rainy	P	I + greenness + brightness + wetness	996.1	385	1.20	−494.03
Summer rainy	P	I + brightness + wetness	997.5	386	1.22	−495.74
Summer rainy	P	I + brightness	997.2	387	1.22	−496.60
Summer rainy	NB	I + greenness + brightness + wetness + surface temperature	989.5	384	0.98	−488.74
Summer rainy	NB	I + greenness + brightness + wetness	988.0	385	0.98	−488.98
Summer rainy	NB	I + brightness + wetness	988.7	386	0.98	−490.34
**Summer rainy**	**NB**	**I + brightness**	**988.0**	**387**	**0.98**	**−491.00**

*Note*: For all models in the table, the response variable was individual fitness as measured by number of offspring produced that survived to age 1. The final model selected for each time interval is bolded. In all cases, negative binomial was a better fit than Poisson with the same predictor variables, according to a likelihood ratio test for each final model.

**TABLE A4:** Summary of all negative binomial models tested using backward stepwise regression for each time interval with female ID included as a random effect.

Time interval	Model	AIC	Residual df	Dispersion value	Log‐likelihood
Annual	I + greenness + brightness + wetness + surface temperature + (1 | female ID)	2460.0	737	0.946	−1223.0
Annual	I + greenness + brightness + surface temperature + (1 | female ID)	2458.4	738	0.942	−1223.2
**Annual**	**I + brightness + surface temperature + (1 | female ID)**	**2456.6**	**739**	**0.940**	**−1223.3**
Annual	I + brightness + surface temperature	2455.5	740	0.871	−1223.7
Summer rainy	I + greenness + brightness + wetness + surface temperature + (1 | female ID)	2455.4	737	1.000	−1220.7
Summer rainy	I + brightness + wetness + surface temperature + (1 | female ID)	2453.4	738	1.000	−1220.7
Summer rainy	I + brightness + surface temperature + (1 | female ID)	2453.9	739	0.978	−1222.0
**Summer rainy**	**I + brightness + (1 | female ID)**	**2453.5**	**740**	**0.953**	**−1222.7**
Summer rainy	I + brightness	2452.6	741	0.872	−1223.3
Winter rainy	I + greenness + brightness + wetness + surface temperature + (1 | female ID)	2456.8	737	0.971	−1221.4
Winter rainy	I + brightness + wetness + surface temperature + (1 | female ID)	2454.9	738	0.972	−1221.4
**Winter rainy**	**I + wetness + surface temperature + (1 | female ID)**	**2454.6**	**739**	**0.960**	**−1222.3**
Winter rainy	I + wetness + surface temperature	2453.6	740	0.875	−1222.8

*Note*: For all models in the table, the response variable was individual fitness as measured by number of offspring produced. The final model selected for each time interval is bolded. The corresponding model without the random variable included is provided in the row following each final model. For all time intervals, the final model retains the same fixed effects as in the final corresponding model without female ID included as a random variable.
